# Protein calorie malnutrition, nutritional intervention and personalized cancer care

**DOI:** 10.18632/oncotarget.15103

**Published:** 2017-02-04

**Authors:** Anju Gangadharan, Sung Eun Choi, Ahmed Hassan, Nehad M. Ayoub, Gina Durante, Sakshi Balwani, Young Hee Kim, Andrew Pecora, Andre Goy, K. Stephen Suh

**Affiliations:** ^1^ The Genomics and Biomarkers Program, JT Cancer Center, Hackensack University Medical Center, Hackensack Meridian Health, Hackensack, NJ, USA; ^2^ Department of Family, Nutrition, and Exercise Sciences, Queens College, The City University of New York, Flushing, NY, USA; ^3^ Department of Clinical Pharmacy, Faculty of Pharmacy, Jordan University of Science and Technology, Irbid, Jordan; ^4^ Department of Clinical Nutrition, Baystate Medical Center, Springfield, MA, USA; ^5^ Clinical Divisions, JT Cancer Center, Hackensack University Medical Center, Hackensack Meridian Health, Hackensack, NJ, USA

**Keywords:** malnutrition, cancer therapy, chemo treatment, biomarkers, nutritional intervention

## Abstract

Cancer patients often experience weight loss caused by protein calorie malnutrition (PCM) during the course of the disease or treatment. PCM is expressed as severe if the patient has two or more of the following characteristics: obvious significant muscle wasting, loss of subcutaneous fat; nutritional intake of <50% of recommended intake for 2 weeks or more; bedridden or otherwise significantly reduced functional capacity; weight loss of >2% in 1 week, 5% in 1 month, or 7.5% in 3 months. Cancer anorexia-cachexia syndrome (CACS) is a multifactorial condition of advanced PCM associated with underlying illness (in this case cancer) and is characterized by loss of muscle with or without loss of fat mass. Cachexia is defined as weight loss of more than 5% of body weight in 12 months or less in the presence of chronic disease. Hence with a chronic illness on board even a small amount of weight loss can open the door to cachexia. These nutritional challenges can lead to severe morbidity and mortality in cancer patients. In the clinic, the application of personalized medicine and the ability to withstand the toxic effects of anti-cancer therapies can be optimized when the patient is in nutritional homeostasis and is free of anorexia and cachexia. Routine assessment of nutritional status and appropriate intervention are essential components of the effort to alleviate effects of malnutrition on quality of life and survival of patients.

## INTRODUCTION

Protein-Calorie Malnutrition (PCM) refers to a nutritional status in which reduced availability of nutrients leads to changes in body composition and function [1]. Disease-associated malnutrition is a common problem among patients with cancer, affecting more than 50% of patients with certain cancers (e.g., pancreas, esophageal, gastrointestinal, and head and neck cancers). Acute and chronic inflammation play a major role in the pathogenesis of cancer-related malnutrition [2]. Altered nutritional status may be due to increased nutrient requirements of the tumor, changes in host metabolism induced by tumor or due to side effects of aggressive anti-cancer therapies [3]. PCM in cancer patients is caused by several factors including loss of appetite, altered taste, and smell, physical inability to ingest food and metabolic alterations including insulin resistance, glucose intolerance, energy imbalance and increased lipolysis and proteolysis. These factors are influenced by the type of cancer, local tumor effects, the anticancer therapy being employed, and psychosocial response to therapy [4, 5].

Weight loss in cancer patients is often characterized by loss of muscle mass and adipose tissue which is different from starvation induced weight loss [6]. If left untreated, it often progresses to severe wasting associated with cancer anorexia-cachexia syndrome (CACS). CACS, a condition of advanced PCM, is a major paraneoplastic syndrome characterized by metabolic abnormalities and loss of skeletal muscle with or without loss of adipose tissues. Anorexia, clinically defined as a loss of appetite or desire to eat is present in 15-20% of cancer patients at diagnosis, and is a common side effect in individuals with metastatic disease [7]. Anorexia is a major component of cachexia.

Cancer cachexia is clinically categorized by severe loss of skeletal muscle and overall - body mass due to metabolic alterations and advanced malnutrition. Cachexia is defined as weight loss of more than 5% of body weight in 12 months or less in the presence of chronic illness; cachexia is also defined as a body mass index (BMI) less than 20 kg/m^2^ accompanied by three of following criteria: decreased muscle strength, fatigue, anorexia, low fat-free mass index, increased levels of C-reactive protein or IL-6 and low serum albumin [8]. The European Society for Clinical Nutrition and Metabolism (ESPEN) identifies pre-cachexia in cancer patients as unintentional weight loss of 5% within six months [7]. The underlying mechanisms of CACS are currently under extensive investigations and there are no clinically available biomarkers that would identify patients who are at a high risk of developing CACS.

Nutrient intake in cancer patients is also affected by abnormalities in gastrointestinal (GI) tract functions due to anticancer therapies. Commonly found symptoms involved in GI tract problems include nausea, vomiting, constipation and diarrhea. Tumor-associated and therapy-associated pain and fatigue also adversely affect nutrient intake [9]. The extent and range of symptoms varies among patients, and there are no readily available clinical methods to identify patients who are more likely to develop serious complications. The American Society for Parenteral and Enteral Nutrition (ASPEN), the American Dietetic Association, and ESPEN suggest that physicians begin nutritional support in malnourished patients and in patients who may have difficulty eating [10–12]. The National Comprehensive Cancer Network (NCCN) suggests treating malnutrition in patients with life expectancies measured in months to years but not in patients with shorter life expectancies [12]. Here, we provide a comprehensive overview of factors affecting nutrient intake in cancer patients with a focus on nutritional support available for undernourished patients; we discuss promising biomarkers under investigation that may be used to identify patients who are likely to develop severe nutritional complications.

## TUMOR-INDUCED MOLECULAR CHANGES ASSOCIATED WITH CACS

**Figure 1:**
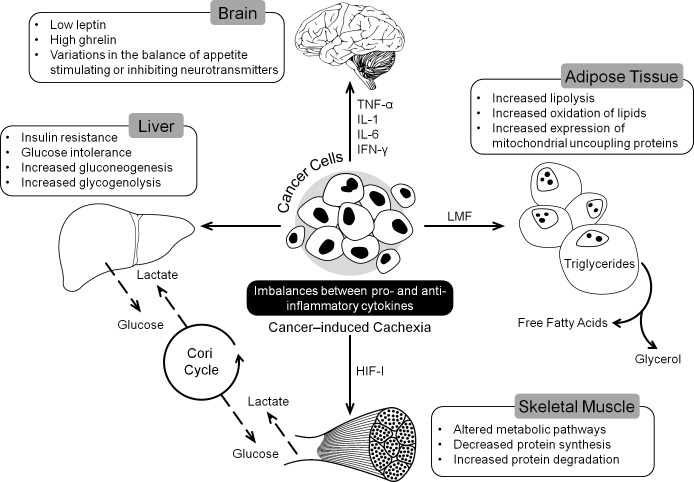
Tumor induced changes in different organs leading to the development of CACS CACS is a multi-organ syndrome promoted by different factors secreted by tumor like cytokines and Lipid Mobilizing Factor (LMF). These factors induce changes in metabolic pathways and variations in appetite ultimately leading to loss of skeletal muscle mass.

The beginnings of malnutrition in cancer patients can be traced to molecular changes induced by tumor-host interactions (Figure 1). Tumor cells have an elevated requirement for nutrients compared to normal tissues. Mobilization of metabolites by tumor, aimed at supporting its growth has a systemic effect on metabolism at the whole organism level which leads to the onset of CACS [13]. CACS occurs as a result of a number of factors including mechanical changes due to the tumor location, systemic inflammation resulting in altered catabolism, and anorexia and neuroendocrine changes occurring due to tumor presence. Skeletal muscle and fat loss in cancer patients caused by decreased protein synthesis, increased protein degradation, and increased lipolysis are not readily reversed through conventional nutritional support [14, 15]. Increase in circulating inflammatory cytokines is implicated in regulating metabolic responses leading to both cachexia and associated anorexia [16].

### Anorexia

Cancer anorexia involves alterations in signaling pathways modulating energy intake mediated by hormones (e.g., leptin), neuropeptides (e.g., Neuropeptide Y [NPY]), inflammatory cytokines (e.g., interleukin-1 [IL-1], interleukin-6 [IL-6], tumor necrosis factor-alpha [TNF- α], and neurotransmitters (e.g., serotonin and dopamine) [17]. Peripheral signals including hormones and inflammatory cytokines being sent to the arcuate nucleus of hypothalamus cause variations in the balance of appetite stimulating neurotransmitters (e.g., NPY and Agouti Related Peptide [AGRP]) or appetite inhibiting neurotransmitters (Opiomelanocortin and Cocaine Amphetamine Related Factor [18]), which alter food intake [19, 20]. It has been reported that in patients with CACS, the melanocortin system is persistently activated [9]. Frequently used tools to diagnose anorexia in cancer patients such as the Functional Assessment of Anorexia/Cachexia Therapy (FAACT) and the North Center Cancer Treatment Group (NCCTG) questionnaire are based on assessments of appetite and appetite related symptoms. However, anorexia is often under diagnosed, and the associated advancement of malnutrition effects become distinguishing features of cachexia [7].

### Inflammatory responses

Pro-inflammatory cytokine activity increases during cancer progression, and systemic inflammation is the hallmark of cancer cachexia indicated by production of acute-phase response (APR) proteins such as C-reactive protein and fibrinogen. Increased production of APRs could result in a higher requirement for amino acids compensated by increase in muscle catabolism [21]. Cachexia often appears with anorexia as a result of imbalances between pro-inflammatory (e.g., TNF-α, IL-1, IL-6, interferon-gamma [IFN-γ]) and anti-inflammatory cytokines (e.g., interleukin-4 [IL-4], interleukin-12 [IL-12], interleukin-15 [IL-15]) [7]. Pro-inflammatory cytokines such as TNF-α, IL-1, IL-6, and IFN-γ are transported across the blood-brain barrier, and they interact with brain endothelial cells causing release of substances that affect appetite (Figure 1). These cytokines alter metabolic pathways in muscle, liver, and adipose tissue, which collectively contribute to cancer cachexia [22]. TNF-α has been implicated in wide range of cachexia- associated mechanisms including protein and lipid synthesis and degradation, gluconeogenesis and expression of Uncoupling proteins. It promotes skeletal muscle wasting by activating the NF-κB pathway [23]. Circulating levels of IL-6 has been shown to be elevated in cachectic cancer patients and it can activate signal transducer and activator of transcription3 (STAT3) which is known to be involved in muscle wasting [24]. Leukemia inhibitory factor, a member of IL-6 family, has also been implicated in pathogenesis of cachexia [25]. However, unlike anorexic patients, cachectic patients have both low leptin (inhibits appetite) levels and high ghrelin (stimulates appetite) levels [26, 27].

### Metabolic alterations

Increased resting energy expenditure and a wide range of metabolic activity from hypo- to hypermetabolism have been reported in cancer patients [28]. Hypermetabolism in malnourished patients contributes to a negative energy balance, which manifests in weight loss. Resting energy expenditure has been shown to vary by tumor type. Gastric and colorectal cancer patients tend to have normal resting energy expenditure, while pancreatic and lung cancer patients have higher resting energy expenditure [29]. The increase in resting energy expenditure experienced by lung cancer patients is generally the result of a systemic inflammatory response [30].

Increased glucose oxidation, protein turn over and lipolysis are widely observed metabolic disturbances in cancer patients. Altered carbohydrate metabolism in cachexia is characterized by insulin resistance, glucose intolerance, and gluconeogenesis from amino acids and lactate [31]. Increased energy consumption in cancer patients due to alterations in glucose metabolism has been reported. Tumor suppressors, p53 and Vhl, which increase dependence on glucose, are often lost in cancer patients [32, 33]. In addition, the hypoxic environment of the tumor and hypoxia-inducible factor I induce the formation of lactate [34]. Excess lactate can enter the Cori cycle in the liver and be converted back to glucose, increasing net ATP consumption (Figure 1). This has been documented in weight loss occurring cancer patients [35]. It has also been reported that gluconeogenesis and glycogenolysis are increased in cancer patients who experience weight loss [36]. The change in glucose level in cancer cells has been estimated to account for 40% of the increased energy expenditure in metastatic cancer [31].

During cachexia, the balance between muscle protein catabolism and anabolism is disrupted. Decrease in levels of anabolic factors and increase in catabolic factors have been observed in patients and cachectic mouse models. Circulating levels of insulin-like growth factor-1(IGF-1), an anabolic factor is decreased in cachectic patients [37]. Expression and production of a negative regulator of IGF-1 and insulin signaling, Impl2, has been observed in different tumor types. This can lead to insulin resistance and tissue wasting [38]. Factors that are upregulated during muscle atrophy include cytokines, activin A, myostatin and TNF-α receptor adapter protein6 (TRAF6) [39–41]. Different proteolytic pathways in the muscle including the ubiquitin-proteasome and autophagy dependent pathways are found to be upregulated in cachectic patients. Mitochondrial metabolism in cachectic muscles is also affected. Uncoupling proteins that reduce proton gradient, produce reactive oxygen species and promote thermogenesis are overexpressed in muscle tissue [42].

Tumors can also cause increased mobilization and oxidation of lipids that result in increased energy expenditure (Figure 1). Lipid mobilizing factor, produced by cachexia-inducing tumors, causes the release of free fatty acids and glycerol from adipose tissue [43]. Lipolysis is also induced by IL-1, IL-6, TNF-α and TNF-γ [43, 44]. Cancer patients exhibit increased expression of mitochondrial uncoupling proteins in brown adipose tissue. Increased expression of uncoupling protein-1 in brown fat results in the loss of proton gradient, which causes uncoupling of ATP synthesis and oxygen consumption leading to increased metabolism and generation of heat. High uncoupling protein-3 mRNA levels are observed in gastrointestinal patients experiencing weight loss [45]. Studies in a knockout mouse model have indicated that activin signaling may play a role in controlling mitochondrial uncoupling and energy expenditure [46].

## NUTRITIONAL COMPLICATIONS ARISING FROM ANTI-CANCER THERAPIES

Patients suffering from cancer and/or receiving anticancer therapy experience a variety of physiological manifestations that negatively affect appetite and dietary intake (Table 1). These side effects can result in reduced food intake, improper digestion, and nutrient absorption leading to the development of anorexia.

**Table 1:** Common anticancer therapy derived complications in advanced cancers

Anti-cancer therapy	Site/Agents	CACS precursors	Ref
Surgery	Oral cavity, larynx, pharynx	Dysphagia, Xerostomia	[48, 49]
	Thoracic, esophagus	Dysphagia, Dumping Syndrome, Pain during alimentation, Weight Loss.	[52]
	Stomach	Dumping Syndrome, Malabsorption (fat, iron, calcium, vitamin B12), Weight Loss	[9, 54, 56]
	Small Intestine (total or subtotal resection)	Malabsorption, Diarrhea, Bacterial Overgrowth	[9]
	Colon	Water and electrolyte loss	[52]
	Pancreas	Malabsorption, Nausea, Vomiting	[57–59]
	Liver	Transitory hypo-albuminemia	[52]
Chemotherapy	Alkylating Agents	Nausea, Vomiting, Mucositis, Stomatitis, Esophagitis, Diarrhea, Malabsorption	[60]
	Antimetabolites	Nausea, Vomiting, Diarrhea Loss of appetite, Constipation.	[203, 204]
	Topoisomerase inhibitors	Diarrhea	[9]
	Corticosteroids	Electrolyte Abnormalities, Hyperglycemia, Pancreatitis	[205]
Radiotherapy Radiotherapy	Total Body	Nausea, Vomiting	[9]
	Head and Neck	Mucositis, Odynofagia, Dysguesia, Dysosmia, Dental Caries, Trismus, Tissue Ulceration, Xerostomia Dysgeusia	[90]
	Esophagus	Reflux, Dysphagia, Odynophagia, Fibrosis, Stenosis Fistula	[88]
	Lung	Odynophagia, Nausea, Fibrosis	[9, 88]
	Abdomen and pelvis	Vomiting, Diarrhea, Acute Enteritis, Colitis, Inflammation and blockage of the intestine or rectum, Ulcer, Malabsorption	[89, 135, 161, 163]
Immunotherapy	Monoclonal Antibodies	Nausea, Vomiting, Diarrhea Toxicity to normal tissues (trastuzumab), induction of severe autoimmunity (ipilimumab)	[91]
	Cytokines	Nausea, Vomiting	[92]
	Immune System Checkpoint Targets	Diarrhea	[91]
Hematopoietic and Peripheral Blood stem cell transplantation	Mouth (GVHD complications) Graft –versus –Host Disease	Mucositis, Eythema, Pain, Xerostomia, Ulcers, Mucocele Restriction of mouth opening from sclerosis	[93]
	GI Tract (GVHD complications)	Nausea, Vomiting, Weight loss	[93]

### Surgery

Efforts to correct nutritional deficiencies should be made prior to surgery to improve the rate of treatment success, however this may be difficult to accomplish if surgery is adjuvant to chemoradiation treatment. Nutritional complications of surgery vary by cancer site. Oral and esophageal resections often cause reduced food consumption and increased nutritional requirements that persist long after surgery [47]. Mastication complications may continue for a year in as many as 50% of post-surgery patients [48, 49]. Surgical intervention on the tongue, salivary glands, or olfactory nerves can cause loss of taste and smell, which can lead to reduced food intake and negative nutritional effects [47]. As many as 50% of pharyngo-laryngectomy and 42% of laryngectomy patients experience dysphagia and chronic xerostomia for three to five years post-surgery [50, 51]. As many as 50-60% of patients undergoing esophagectomy experience loss of hunger, which is accompanied by reflux; 75% suffer from postprandial dumping syndrome, and 80-90% experience early satiety. Associated symptoms include dysphagia, weight loss, increased stool frequency, and dumping syndrome which consists of abdominal cramps, nausea, dizziness, diarrhea, and diaphoresis (Table 1) [52].

Surgeries of the gastrointestinal tract result in alterations to digestion and absorption of nutrients. Akin to head and neck surgeries, dysgeusia and dysosmia have been reported to occur in half of all patients undergoing upper gastrointestinal surgery; symptoms generally resolve within a 6- to 12-month timeframe [53]. However, 80% of cancer patients undergoing gastrectomy are malnourished due to maldigestion, malabsorption, shortened intestinal transit time, and bacterial overgrowth [54, 55]. Factors that influence maldigestion and malabsorption include lack of gastric hormones (partly caused by vagotomy) and the body's inability to stimulate biliary and pancreatic secretions [56].

Malnutrition subsequent to pancreatico-duodenectomy is often an outcome of disease progression, extent of parenchymal resection, and functional status of the remaining pancreas [57, 58]. Within two years post-surgery, more than 50% of patients develop pancreatic exocrine insufficiency. The loss of enzymatic activity in the intestinal lumen, particularly loss of lipase activity, results in malabsorption of fat, protein, starch, and fat-soluble vitamins, such as A, D, E, and K [59].

### Chemotherapy

Toxicity of chemotherapy induces a host of complications in cancer patients including nausea, vomiting, anorexia, taste and smell changes, early satiety, mucositis, esophagitis, diarrhea, xerostomia, and constipation (Table 1). These symptoms occur as a function of the length and number of treatments. One of the most notable features of chemotherapy is nausea, which occurs in 84% of patients. Nausea occurring within 24 hrs of treatment is mediated by activation of serotonin type 3 receptors, while delayed symptoms involve several factors including adrenal hormones, substance P, and gastrointestinal motility disruption [9].

In addition to decreased nutrient intake, metabolic abnormalities such as hyperglycemia and hypercalcemia occur post chemotherapy [53]. Alkylating agents such as cyclophosphamide, ifosfamide, or methotrexate can cause malabsorption by inducing direct mucosal and metabolic alterations [60]. Due to alterations from mucositis, erosive lesions may appear in the gastrointestinal tract [53]. Antineoplastic agents such as fluorouracil, doxorubicin, methotrexate, and cisplatin can induce severe gastrointestinal complications [61]. Biological therapies with interferons or with monoclonal antibodies, such as bevacizumab or cetuximab, can cause low to moderate nausea and/or vomiting. Tyrosine kinase inhibitors, such as lapatinib cause diarrhea [9].

Taste changes are common, and occur in 45-84% of cancer patients receiving chemotherapy [62–66]; changes in the ability to smell occur in 5% to 60% of patients [67–69]. Patients with head and neck tumors have an especially high prevalence of chemosensory disorders [70, 71] due to the location of their cancer and the long-term effects of cancer therapies [72]. Chemosensory dysfunctions affect food intake and appetite [73–75] leading to the development of food aversions and weight loss [76, 77]. Taste changes have been assessed by electrogustometry [78–80], which involves the application of an electric current to the tongue. Berteretche et al found an increase in electrical taste detection thresholds in chemotherapy patients compared to healthy controls [62]. Ovesen et al. observed decreased electrical taste detection thresholds in patients with different solid tumors undergoing chemotherapy compared to control patients with non-cancerous disease [81]. In lung cancer patients and treatment responders, multiple studies have reported a decrease in electrical taste detection thresholds post chemotherapy compared to pre-chemotherapy [79, 81].

Chemotherapy patients showed increased chemical taste thresholds assessed by application of dilutions of basic taste substances [67, 79, 80, 82, 83] or with impregnated filter paper Taste Strips [84]. Higher thresholds were found for bitter [85, 86], sweet [86, 87], sour [84, 86], and salty tastes [86]. However, in other studies changes or variations in sensitivity to different concentrations were not observed [82, 83]. Because these studies involved small sample sizes, these contradictory findings might be artefactual.

Few studies have prospectively investigated smell alterations in chemotherapy patients. However, multiple studies have reported that smell thresholds were unchanged during chemotherapy [69, 81]. Yakirevitch et al. observed that smell thresholds were increased after the end of treatment, although there was no change during chemotherapy [69]. Steinbach et al. also reported a decrease in olfactory function during chemotherapy, and found that compared to smell thresholds, smell discrimination and identification were largely unaffected [84].

### Radiotherapy

The side effects of radiation therapy depend on the area irradiated, total dose, fractionation, duration, and volume irradiated. Side effects last several weeks; patients only begin the process of healing after the first 2-3 weeks out from treatment due to the continued radiation effects. Because of this,, 90% of patients that undergo irradiation for head and neck, thoracic, abdominal, and pelvic tumors become malnourished and experience weight loss (Table 1) [9]. Radiotherapy of the head, neck, and thorax can cause xerostomia, mucositis, hypophagia, and pain. Radiation treatment of the oral cavity causes nausea, vomiting, dysphagia, odynophagia, sore throat, anorexia, esophagitis, mucositis, xerostomia, tissue ulceration, and taste alterations [9]. Oral cavity irradiation can have direct toxic effects on taste buds through effects on innervating nerve fibers, and can affect secretary cell functions in the mouth, reducing output and altering viscosity of saliva [53]. Acute esophagitis is a common side effect of radiotherapies for intra-thoracic neoplasms following chemotherapy or of radiotherapy exceeding 50 Gy, which is the case for 30% of cancer patients [88].

Abdominal irradiation often causes malabsorption because of direct toxic effects on microvilli of the gastrointestinal mucosa. Nausea or emesis occurring around three days post-irradiation is reported in approximately 50% of patients receiving upper abdominal radiation [9, 89]. Radiation induced emesis occurs in more than 90% of patients receiving total body irradiation [9].

Chronic radiation enteropathy is another complication that has a strong negative effect on the nutritional status of cancer patients due to the formation of multiple severe gastrointestinal strictures and fistulas [47]. In the chronic phase, when a stenosis occurs following esophagitis, the most common intervention is the initiation of enteral nutrition; otherwise, esophageal dilation and stent placement are necessary [9]. Symptoms are usually most severe in the week following radiation, but generally plateau and may wither thereafter [90]. The pain caused by these side effects may be severe enough to limit adequate hydration and nutrition [9].

### Immunotherapy

Depending on the specific therapy used, immunotherapy can cause gradual or drastic weight loss. Monoclonal antibodies, which are used to block cancer-cell receptors for growth-stimulating factors, may cause a cascade of symptoms due to their inherent toxicity and possible autoimmune manifestations [91]. Cytokine therapy involves the therapeutic introduction of cytokines to induce the immune response to disrupt tumor growth. Treatments with cytokines such as interleukin 2 or granulocyte-macrophage colony stimulating factor produce side effects including weight loss, fever, nausea, vomiting, and diarrhea (Table 1) [92].

### Hematopoietic and peripheral blood stem cell transplantation(HSCT)

Patients receiving allogeneic donor stem cells often develop mucositis depending on the level of cytotoxicity of the immunosuppressive drug regimen, and may have difficulty meeting caloric needs [93]. Often the nutritional deficiencies result from acute graft *versus* host disease (GVHD), especially of the alimentary tract, resulting in taste changes, oral dryness, thick saliva, mouth and throat sores, nausea and vomiting, diarrhea, constipation, lack of appetite/weight loss, and weight gain [93].

## NUTRITIONAL SCREENING IN CANCER PATIENTS

Patients at high risk of developing PCM can be identified using simple, non-invasive tools, which are sufficiently sensitive to predict nutritional deterioration under current and likely future circumstances associated with disease processes. Thus, identified high-risk patients should be seen by an expert for detailed assessment of metabolism, body composition, and other variables to determine an appropriate nutritional therapy regimen. Nutritional screening should ideally be done before the start of anti-cancer therapy so that the nutritionist and dietician can give informed dietary recommendations according to scheduled treatment. Three commonly used nutritional screening tools used in assessment of oncology patients are Nutritional Risk Screening (NRS-2002), Malnutrition Screening Tool (MST), and the more detailed Patient-Generated Subjective Global Assessment (PG-SGA) [94].

NRS-2002 generates a nutritional score based on change in dietary intake, recent weight loss, BMI changes, and a subjective assessment of disease severity[95]. NRS-2002 is widely used in evaluating hospitalized patients, and is a good predictor of post-operative complications in cancer patients [96, 97]. MST is a three question test to assess loss of appetite and recent weight loss in medical, surgical, and oncology patients [94]. MST has been accepted as a predictable test for identifying high-risk oncology outpatients, especially those who are undergoing radiotherapy [98]. Subjective Global Assessment (SGA) is a widely used objective method based on medical history and patient physical examination [99]. PG-SGA has been specifically tailored for cancer patients incorporating an expanded questionnaire and a numerical scoring system [100, 101]. SGA is the most commonly used system for nutritional assessment, and is considered to be the gold standard for evaluating oncology patients.

In patients with increased risk of malnutrition, a detailed evaluation accompanied with data collection is recommended. Information that need to be gathered include demographic and anthropometric data, details on the disease and its treatments and biochemical data which includes the plasma concentrations of positive and negative APR proteins [102]. Although bodyweight is the most important endpoint of any cachexia treatment, any tool used should include measurements for body composition, physical performance status such as Eastern Co-operative Oncology group (ECOG) score for cancer patients and quality of life. Clusters of multiple parameters are recommended for assessment to avoid interference from tumor derived factors[2].

## MANAGEMENT FOR NUTRITION-RELATED COMPLICATIONS

**Figure 2:**
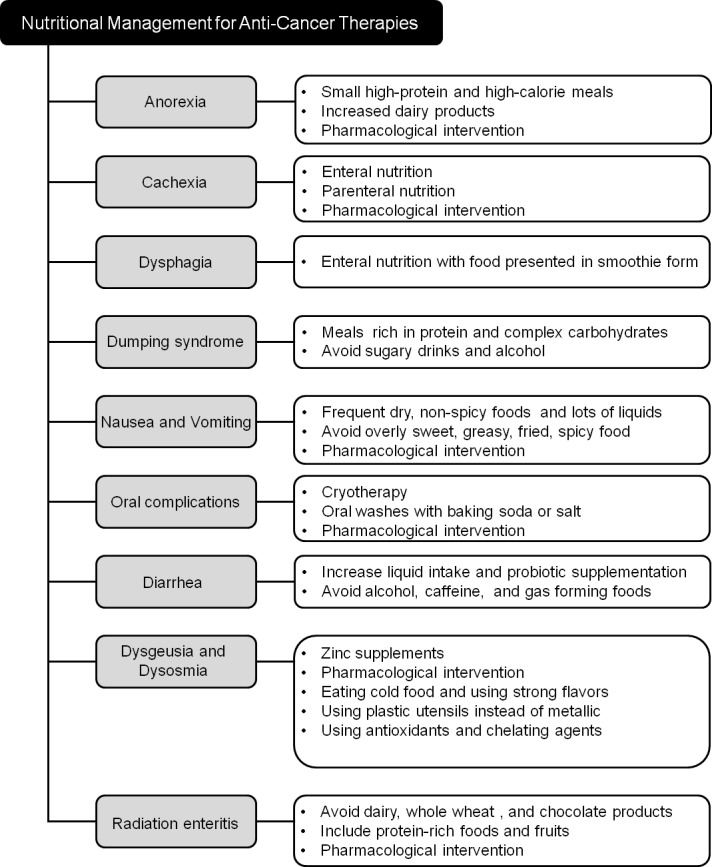
Nutritional support strategies for common physiological complications arising from anti-cancer therapies

Adequate calorie and nutrient intake is imperative for the management of cancer-related symptoms and adverse effects of treatment. In general, the most highly recommended diet for cancer patients is a high calorie, high protein diet that includes a wide variety of fruits, vegetables, and proteins, and is low in saturated fats, but high in monounsaturated fats. Aggressive efforts should be made to ensure that patients continue to consume foods that are high in calories and protein to meet their nutritional needs throughout the course of their treatment (Figure 2).

Due to the complex nature of complications arising from cancer or anti-cancer therapies, a significant effort is necessary to identify drugs that can alleviate the symptoms and provide relief to patients. Although drugs are approved by the FDA for safety, clinical trials focused on assessing effectiveness for treating symptoms have been limited (small patient cohorts, lack of complete patient history). Despite these shortcomings, drugs have been identified that are effective for treating symptoms arising from cancer and anti-cancer therapies (Supplementary Table 1).

### Anorexia

Anorexia is one of the most common problems for cancer patients. To manage anorexia, cancer patients are advised to eat small high-protein and high-calorie meals every 1-2 hours instead of 3 larger meals, and take to liquid supplements (special drinks containing nutrients) [47]. Eating more dairy products also helps to ameliorate anorexia (Figure 2). Also, insure that patient is not suffering with constipation and if so discuss methods for relief as constipation can promote feelings of poor appetite/intake/early satiety and aggravate anorexia.

The most widely employed appetite stimulants used to treat anorexia are Megestrol acetate (Megace) and Medroxyprogesterone acetate (MPA), both of which are synthetic progestins, which stimulate appetite via Neuropeptide Y (NPY)-release in the hypothalamus [103] or by downregulating synthesis and release of proinflammatory cytokines [104]. Anorexia has been shown to be treatable with androgenic and anabolic steroids including nadrolone, decanoate, and oxandrolone [105–107].

### Cachexia

The majority of cancer patients experience weight loss as their disease progresses. Nutritional management of cachexia in cancer patients involves intake of high-protein and high-energy foods. Supplementation with eicosapentaenoic acid, antioxidants, and proteins may reverse severe weight loss in cancer patients with high inflammatory stress [108]. However care has to be taken to avoid interference of the supplements in inflammatory response induced by treatment aimed at destroying cancer cells. Hence antioxidant supplements are only prescribed in practice several months or years post treatment. A combination of ß-hydroxy-ß-methylbutyrate (HMB), arginine, and glutamine has demonstrated an overall benefit including increased lean body mass, improved emotional profile, less weakness, and improved hematological parameters [109].

Although multifactorial, cachexia can be treated with corticosteroids such as dexamethasone, prednisolone, and methylprednisolone that enhance appetite and sensation of well-being [110]. Several neuropeptides regulate appetite, and are currently undergoing clinical trials to determine their efficacy in the treatment of cancer cachexia. Among these is ghrelin, a neuropeptide released from the stomach in response to fasting that stimulates food intake and improves total and lean body mass. Ghrelin has been suggested as a therapeutic approach for the treatment of cachexia [111]. Anamorelin, a ghrelin agonist has completed phase III trials in which it was shown to have a significant impact on reversal of skeletal muscle loss and a positive effect on appetite and weight gain [112]

### Dysphagia

Dysphagia associated with head, neck, and esophageal tumors can persist despite surgical excision of the tumor mass. Mechanical dysphagia can morph into neurogenic dysphagia due to ablations of muscular and nervous tissue, and presence of chemo and/or radiotherapy [113, 114]. Nutritional deficiencies caused by mechanical dysphagia can be avoided by early enteral nutrition [9]. Improving the texture of food presented in smoothie form using food thickeners, diluents, and lubricants is highly recommended. Fats consumed in smaller volumes can increase calorie intake [115].

### Dumping syndrome

Dumping syndrome usually follows gastrectomy and esophagectomy. Early dumping syndrome occurs immediately following meals rich in simple carbohydrates due to rapid abdominal distension with the release of vasoactive substances such as serotonin and bradykinin [9]. This can be avoided by small, frequent meals high in protein and complex carbohydrates, and allowing a 40 minute gap between food and drink ingestion (Figure 2). Complex carbohydrates rich in fiber delay glucose absorption and prolong bowel transit time. Supplementation with dietary fiber, including pectin and guar gum, has been shown to effectively treat hypoglycemia [116]. Late dumping syndrome occurs 2-3 hours following a meal due to a postprandial hypoglycemia crisis that develops due to hyperinsulinemia induced by a rapid influx of glucose into the blood [116]. Avoiding sugar containing drinks and alcohol is also recommended to prevent late dumping [115].

### Nausea and vomiting

Before and during chemotherapy/radiotherapy, patients may experience anticipatory nausea and vomiting. In addition, tastes, smells, visual cues, and thoughts might trigger the onset of emesis [117, 118]. In general, nausea can be avoided by eating dry, non-spicy foods after waking up and every few hours during the day. Foods that are overly sweet, greasy, fried, spicy, or have strong odors should be avoided. Nausea can also be avoided by sitting up or reclining with head raised for at least an hour after eating. Care should be taken to drink sufficient liquids to prevent dehydration. A bad taste in the mouth can be ameliorated by sucking on hard sweet candies or chewing peppermint chewing gum [119]. Avoid hot, stuffy rooms or strong cooking odors by using baking bags as well.

Metoclopramide, Haloperidol, Cyclizine, Domperidone, Ondansetron, Scopolamine, Prochlorperazine, Chlorpromazine, and Levomepromazine have been found to be effective in reducing the severity of nausea and vomiting arising from chemotherapy [120–123]

### Oral complications

Oxidative stress caused by chemotherapy and radiotherapy often leads to inflammation resulting in a host of ulcer-inducing alimentary tract inflammatory symptoms. Mucositis is often related to antimetabolites (e.g., 5-fluorouracil), antitumor antibiotics, taxanes, cisplatin, etoposide, cyclophosphamide, and irinotecan [124, 125]. Oral mucositis develops in a significant number of patients undergoing chemotherapy for different cancers and patients undergoing radiotherapy for head and neck cancers. Pain control, nutritional support, oral decontamination, management of bleeding and dry mouth and therapeutic interventions are used in the clinic to tackle the condition [126]. Cryotherapy is often performed in conjunction with chemotherapy. Oral rinsing with ice water or sucking on ice chips causes local vasoconstriction, which reduces the distribution of drug, reducing the severity of oral mucositis[127–129]. In combination with induced myelosuppression caused by antimetabolites, mucositis can result in viral and bacterial infections. Oral washes with baking soda or salt can be used to prevent infection and to provide soothing effects [129]. Frequent dental examinations decrease the possibility of infection, as well.

Head and neck cancer patients suffering from xerostomia (dry mouth) have shown improvement when treated with Amifostine, Pilocarpine, and Cevimeline, which are cholinergic agonists that work by stimulating certain nerves to increase saliva production [130–132]. In addition, head and neck cancer patients may suffer from mucositis for which analgesic therapy and antibiotics/antifungals are given in addition to drugs such as Palifermin [Keratinocyte growth factor (KGF) produced by recombinant DNA technology] to prevent infection[18]. Mouthwashes containing diphenhydramine, lidocaine, nystatin, and corticosteroids are also used in clinical practice [127, 129].

### Diarrhea

Severe diarrhea is usually treated with a combination of clear liquid intake of at least 3 L per day, water soluble fiber supplements, and anti-diarrheal drugs[133]. It is helpful to eat small, frequent meals, while avoiding alcohol, caffeine, gas-forming food or drink, high fiber foods, high fat foods, and spicy foods. Lactose intolerant patients should avoid lactose-containing foods. Diarrhea resulting from anti-cancer therapies can be treated with Imodium, Cholestyramine, Diphenoxylate, and Octreotide acetate [120–123, 134]. These drugs can also be used in conjunction with anti-inflammatory drugs and antibiotics to treat radiation enteritis [135]. Probiotic supplementation is a potential preventive method for diarrhea resulting from radiotherapy to the lower abdomen and pelvis. In a study of 490 patients receiving surgery for sigmoid, rectal, and cervical cancers, supplementation with VSL#3® (VSL Pharmaceuticals, Inc) before and during subsequent radiation led to a significant difference in the number of bowel movements and toxicity of diarrhea [133]. Supplementation with another potential preventative probiotic, *Lactobacillus acidophilus*, significantly reduced diarrhea in 1748 patients following radiation to the pelvis [136]. In addition, to reduce radiation-induced diarrhea, psyllium fiber supplementation is helpful [137, 138].

### Dysgeusia and dysosmia

Dysgeusia is a distortion of the sense of taste and dysosmia is any alteration of the perception of smell. Dysgeusia is a condition in which a foul, salty, rancid, or metallic taste sensation persists in the mouth and also often associated with ageusia, which is the complete lack of taste, and hypogeusia, which is a decrease in taste sensitivity. Often, people who feel they have a problem with their sense of taste are experiencing a loss of smell instead of a loss of taste [139]. Zinc plays an important role in taste perception, and zinc deficiency is often responsible for taste perception abnormalities in otherwise healthy persons [140], in various diseases [86, 141], and in drug-induced taste disorders [142]. Beneficial effects of zinc sulfate [80, 143, 144] or polaprezinc (zinc-L-carnosine) [145] on taste disturbances have been reported in patients with head and neck cancer or lung cancer (Figure 2). However, a phase III trial found no significant effect of zinc sulfate therapy on the median interval to taste alterations following radiotherapy, and no significant benefit of zinc therapy on taste or smell was found among cancer patients undergoing chemotherapy [146, 147]. Zinc supplementation should be used cautiously until further research confirms its efficacy, because long-term and excessive consumption of zinc may have a negative impact on the immune system in cancer patients [148]. Zinc supplementation should not go on beyond 3 months as iron and copper stores can be lowered.

Drugs such as bovine lactoferrin, which can treat taste alterations, are currently undergoing clinical study. Results of a randomized placebo-controlled pilot study to determine if administration of delta-9-tetrahydrocannabinol (THC) improved taste and smell perception as well as appetite, caloric intake, and quality of life for cancer patients with chemosensory alterations showed that treatment with THC improved chemosensory perception as well as appetite in patients with advanced cancer [149]. THC increases appetite via endocannabinoid receptors (CB1r) [150]. CB1r are located in reward-related areas of brain and in the olfactory epithelium and bulb [151, 152]. CB1r are involved in peripheral odor processing and potentially in taste function [151, 153]. A detailed list of drugs used for treating therapy-related symptoms is provided in Supplementary Table 1.

Metallic taste is frequently reported by cancer patients [154]. After at least two cycles of chemotherapy, 78% (29/37) of patients with various cancer types described their perceived taste change as metallic [64]. Several strategies have been suggested for managing metallic taste. The most commonly mentioned strategy is the use plastic instead of metallic utensils [77, 155]. Patients receiving cyclophosphamide as part of their treatment reported a slight but lingering metallic taste; a few of these patients reported that using plastic utensils made food more palatable [156]. In patients with lymphoma, breast, lung, or ovarian cancer, consumption of cold foods was found to be more helpful for metallic taste than for other taste alterations [64]. In a study of ten colorectal cancer patients treated with oxaliplatin-containing chemotherapy, one patient reduced the metallic taste through the use of very strong flavors, such as lots of salt [157].

A new approach was suggested in a pilot study using the fruit *Synsepalum dulcificum*, also known as “miracle fruit,” to improve food palatability for patients receiving chemotherapy [158]. “Miracle fruit” was developed as a sweetness enhancer. It contains the protein miraculin, which binds to sweet receptors on the tongue, having the effect of turning sour-tasting foods into sweet, and providing short-duration masking of certain unpleasant tastes, including metallic [159]. However larger studies are warranted on possible side effects of using miracle fruit since its antioxidant activity could interfere with the action of some chemotherapeutic drugs such as doxorubicin and platinum compounds. Also, stomach ache and sore throat have been reported with use. Lipid oxidation is thought to play a role in the development of metallic taste. Lipid oxidation may be reduced or prevented by use of antioxidants and chelating agents. A study of the effect of antioxidants (Vitamin C and E) and chelating agents (Ethylenediaminetetraacetic acid and lactoferrin) on the perceived intensity of metallic taste and lipid oxidation in healthy participants (22 participants, age 19-53 years) [160]. The antioxidants were not effective in removing the metallic taste, but the chelating agents were effective; lactoferrin completely eliminated the metallic taste in all participants.

### Radiation enteritis

The use of radiation in the treatment of cancer comes with risks, one of which is radiation enteritis. Radiation enteritis is inflammation of the small and/or large intestine resulting from radiation treatments of the stomach, sexual organs, bladder or rectum. More than one fifth of patients receiving radiotherapy will develop radiation enteritis, with chronic enteritis developing between 18 months and 6 years following treatment [161–163]. It is often suggested that patients affected by radiation enteritis make changes in their diet to reduce aggravation to the digestive system. Among the recommendations are avoidance of dairy products (with exception of yogurt), whole wheat products, chocolate, and raw vegetables, and inclusion of protein-rich foods and fruits in the diet (Figure 2).

### Nutrition support therapy

Moderately to severely malnourished cancer patients who are at risk of not receiving adequate nutrition for 7 to 14 days following surgery would benefit from enteral nutrition (EN). Administration of an immune enhancing formula that includes supplemental arginine, ω-3 fatty acids, and nucleotides has been shown to decrease the incidence of surgical complications in these patients [164]. EN is also the preferred mode of nutritional support in post-operative malnourished patients as it is associated with reduced incidences of infectious complications and hyperglycemia [165]. EN delivered by feeding tube is also used in patients with head and neck cancer undergoing chemoradiotherapy. Depending on the tumor status and the patient's nutritional status, administration of prophylactic or reactive enteral tube feedings is advisable to combat malnourishment [166].

Although oral and EN methods are preferred, parenteral nutrition (PN) is generally used when patients begin to experience severe side effects from anticancer therapies. PN is recommended when malnourished patients are unable to digest and absorb nutrients delivered enterally for more than 7 to 10 days [167]. Cachectic and hypophagic patients suffering from sub-acute intestinal obstruction due to peritoneal carcinomatosis may require long term PN [168]. PN is recommended in cases where severe mucositis or severe radiation enteritis is present, or when oral or enteral nutrition is not possible [169]. PN is not recommended for non-aphagic patients or for those with severe gastrointestinal obstructions, in which case PN is ineffective and possibly damaging but PN is typically recommended or even required for patients with gastrointestinal obstructions; in these cases, some terminal patients (3+ months expected lifespan) palliative PN should be considered, but is not a cancer-specific treatment and will not likely prolong the patient's life [169].

PN will not increase survival in a patient who is undergoing chemotherapy and radiation therapy, and is only recommended for patients whose nutrient absorption is inadequate and EN is not possible [167]. In HSCT patients, PN is not routinely recommended but may be used to ensure nutrition in patients experiencing severe mucositis, ileus, and intractable vomiting [168]. Glutamine supplementation may be beneficial to these patients. PN should be discontinued when patients are able to attain 50% of nutritional needs enterally [168]. If GVHD develops, patients should be put on PN, and are usually not taken off PN until bone marrow recovers [168]. In patients with incurable cancers, PN is recommended when life expectancy is greater than 2 to 3 months, EN is not plausible, PN will likely improve quality of life, and the patient requests this form of nutrition [168]. Both EN and PN involve risks, including psychological risks associated with stress and discomfort from feeding tube insertion. Common complications also include infections, metabolic issues such as hyperglycemia, electrolyte imbalances, diarrhea, and, in the terminally ill, fluid overload [169].

## BIOMARKERS FOR NUTRITIONAL STATUS

Diagnosis and management of CACS is a challenge; identification of biomarkers to precisely diagnose CACS in early stages and to predict the progression and outcome would significantly impact treatment and survival of cancer patients. Here we list a few promising candidate biomarkers with diagnostic and prognostic significance in CACS (Table 2).

**Table 2:** List of studies evaluating candidate biomarkers for CACS and therapy-associated complications

Physiological Conditions	Biomarker	Function	Comparison/ Purpose	Results	Ref
CACS	IL-1β	Pro-inflammatory cytokine	Plasma levels of pro-inflammatory cytokines in advanced cancer patients	IL-1β levels strongly associated with subjective (weight loss, loss of appetite) and objective (albumin and CRP levels) measurements of cancer cachexia.	[206]
	IL-6	Pro-inflammatory cytokine	Identification of high risk factors contributing to increased serum IL-6 levels in chemo-naïve advanced pancreatic cancer patients.	High serum IL-6 related to anemia (P < 0.01), high CRP levels (P = 0.02), severe fatigue (P = 0.02) and hepatic metastasis (P < 0.01).	[207]
			Serum pro-inflammatory cytokine levels in gastroesophageal patients.	IL-6 and other pro-inflammatory markers are elevated in cachetic gastroesophageal patients compared to non-cachetic (6.582pg/ml vs 3.018pg/ml) and healthy controls (6.582pg/ml vs 1.002pg/ml).	[208]
	IL-8	Pro-inflammatory cytokine	Serum IL-8 levels were compared between healthy controls, non-cachetic gastric cancer (GC) patients and cachetic GC patients.	IL-8 levels were significicantly higher in cachetic patients compared to non-cachetic patients and healthy subjects (1.413 ± 0.130 ng/mL vs 0.899 ± 0.076 ng/mL).	[209]
			Serum IL-8 levels in gastroesophageal patients.	IL-8 is elevated in cachetic patients compared to non-cachetic and healthy controls.	[208]
	Ghrelin	Gastric hormone involved in energy balance and hunger.	Role of ghrelin in cachexia and its potential as a diagnostic tool in NSCLC patients.	Serum ghrelin levels were higher in patients experiencing weightloss compared to healthy individuals (0.5 ± 0.4 ng/ml vs. 0.4 ± 0.3ng/ml) and patients without weight loss. (0.56 ± 0.24 ng/ml vs. 0.52 ± 0.44 ng/ml)	[210]
	Leptin	Adipokine involved in energy homeostasis	Predict potential of using leptin as a diagnostic and prognostic biomarker for cachexia in cancer patients.	Serum leptin was significantly lower in cachetic cancer patients with a diagnostic (sensitivity 79%, specificity 73%) and prognostic significance (HR 0.94; 95% CI 0.92 - 0.96; p < 0.0001).	[171]
	Angiotensin II	Peptide hormone involved in skeletal muscle maintenance.	Identification of blood based biomarkers of cachexia in advanced cancer patients.	Pre-cachetic (~18±2pg/ml) and cachetic (~17±2pg/ml ) patients showed significantly elevated levels of angiotensin II in blood compared to non-cachetic (~8±1.8pg/ml) patients.	[179]
	Growth Differentiation Factor (GDF-15)	TGFβ family cytokine	Measurement of plasma GDF-15 levels in cancer patients compared to controls	Plasma GDF-15 levels were elveated in cancer patients experiencing weight loss compared to those who are not.(median levels of GDF-15- 2.5ng/ml compared to 1.5ng/ml)	[188]
Nausea and Vomiting	Arginine Vasopressin^202^	Direct stimulation of the medullary vomiting center via vagal afferents	AVP levels in plasma of patients with vomiting after chemotherapy to those who don't.	Increased levels of AVP in patients with vomiting symptoms(4 to 129 fold difference).	[193]
Mucositis	Citrulline	Inflammation caused by GI tract damage.	Usage of citrulline as a marker for GI mucositis in HSCT patients	Citrulline levels were <10μmol/L in patients with severe mucositis.	[196]
Chemotherapy – induced Diarrhea	Calprotectin	Calcium binding protein	Identification of biomarkers for chemotherapy-induced diarrhea in fecal samples of cancer patients	Fecal calprotectin levels were increased in patients with diarrhea (6.82±1.20 ng/ml)compared to healthy controls( 0.54±0.54 ng/ml).	[197]
Radiation-induced Acute Intestinal Symptoms (diarrhea, tenesmus, bloody stools)	Transthyretin	Thyroid hormone-binding protein	Identification of biomarkers for the condition in cervical cancer patients.	Patients with RIAIS showed significantly decreased levels of serum TTR compared to patients without RIAIS ( 22.112 vs 27.528 ng/ml)	[200]
	Neurosecretory protein VGF fragments	Energy homeostasis	Identification of biomarkers for the condition in cervical cancer patients.	Patients with RIAIS showed significantly higher levels of serum TTR compared to patients without RIAIS ( 3824.244 vs 2944.890 pg/ml)	[200]
GVHD	Regenerating islet-derived 3-alpha (REG3α)	Protect epithelial barrier function of intestinal mucosa	Identification of plasma biomarkers specifically for gastrointestinal GVHD.	REG3α levels showed 3 fold increase in patients with GI GVHD and serves as a prognostic indicator for non-relapse mortality.	[211]

### Ghrelin

Ghrelin is a circulating, gut-derived hormone that crosses the blood brain barrier, acts through growth hormone secretagogue receptor in several parts of the brain to stimulate appetite and increase food intake [170]. Given the significance of ghrelin in increasing food intake, multiple studies have looked at plasma or serum ghrelin levels in cancer patients. Ghrelin levels were elevated in 140 cachectic adults with different cancers compared to healthy controls [171]. Individual studies have also reported elevated ghrelin levels in cachectic patients with lung or colon cancer [27, 172, 173]. Biologically active octanoylated ghrelin and active to total ghrelin levels have also been found to be increased in cancer patients compared to non-cancer controls [174]. It has been suggested that increased ghrelin levels might be a compensatory response to weight loss. Lower ghrelin levels in cancer patients have also been linked to increased survival [171]. Detailed studies on regulatory mechanisms of production and secretion of total and active ghrelin levels in cancer patients at risk of developing cachexia are needed to evaluate the potential of ghrelin as a biomarker for cachexia.

### Leptin

Leptin is produced predominantly in adipose tissues, and is a pleiotropic cytokine involved in the regulation of energy homeostasis, metabolism, and immune function. Leptin receptor is expressed in several cancers, and leptin signaling has been proposed to be involved in carcinogenesis [175]. Plasma leptin concentration is dependent on the amount of fat tissue. Lung and gastrointestinal cancer cachectic patients show lower serum leptin levels, presumably due to decreased fat. However, leptin levels have been found to be elevated in breast and gynecological cancer patients due to factors other than cachexia, limiting the potential of leptin as a universal biomarker for identifying cachectic patients [43]. However, additional studies on the role of leptin in cachexia and use of leptin as a biomarker are warranted.

### Angiotensin II

Angiotensin II, the main component of the renin-angiotensin system (RAS), increases protein degradation and apoptosis in skeletal muscle [176]. Angiotensin II inhibits the IGF-1 signaling pathway, which results in decreased protein synthesis. Increased angiotensin levels also cause elevated levels of TNF-α, IL-6, and myostatin, which results in increased protein degradation and attenuated protein synthesis. In addition, angiotensin II regulates anorexic/orexic associated neuropeptides to regulate appetite and food intake [177, 178]. Angiotensin II mRNA in blood and plasma levels of angiotensin II were found to be elevated in patients with different cancer types prior to anti-cancer therapy. Angiotensin II was found to be elevated in both pre-cachectic and cachectic cancer patients compared to patients without cachexia, suggesting that angiotensin II has potential as a biomarker for early detection of patients at increased risk [179]. Angiotensin converting enzyme inhibitors have been used to inhibit muscle wasting and improve weight loss, which further indicates the possible significance of angiotensin in management of cachexia [180].

### Cytokines

Systemic inflammation is closely associated with development of CACS, and pro-inflammatory cytokines have been explored as potential biomarkers for cachexia (Table 2). Plasma levels of several pro-inflammatory cytokines including TNFα, IL-6, IL-1 and IFN-γ in circulation have been assessed as markers of cachexia [181–184]. Although levels of these cytokines were found to be elevated in multiple studies, the results were not consistent [185, 186]. Variations in assay sensitivities, short half-life of cytokines, and presence of natural cytokine inhibitors may be associated with differences in detected levels of cytokines [183]. The TGFβ signaling pathway has been implicated in development of cachexia, and its superfamily members including TGFβ-1 and Growth Differentiation Factor (GDF-15) have been found to be elevated in plasma of pre-cachectic and cachectic cancer patients experiencing weight loss [179, 187, 188].

### MicroRNAs (miRNAs)

Recently, several miRNAs, short non-coding RNAs that play a significant regulatory role in several biological processes, have been found to play important roles in muscle wasting processes. Lung and pancreatic cancer-derived microvesicles expressing miR-21 were shown to activate TLR7 receptor and to induce apoptosis of skeletal muscle cells [189]. Global miRNA profiling in adipose tissues of gastrointestinal cancer patients with or without cachexia indicated that miR-483-5p/-23a/-744/-99b were downregulated and that miR-378 was up regulated in cachexia. Upregulation of miR378 correlated with catecholamine-stimulated lipolysis in adipocytes and with expression of key lipolytic regulators [190]. These findings indicate a key regulatory role for miRNAs in cancer cachexia, and suggest that, with further analysis, miRNAs may provide useful diagnostic and prognostic markers.

## BIOMARKERS FOR ANTI-CANCER THERAPY-RELATED SYMPTOMS

Identification of biomarkers that predict the likelihood of occurrence of particular physiological complications following cancer therapy is difficult, in part, because of the complexity of generating an accessible biomarker that accurately predicts outcomes under highly variable circumstances such as age, nutritional and health status, medication, metabolic activity, and therapeutic modality [191]. These issues can be complicated further by overlapping symptoms that may be regulated by similar physiological underpinnings [192]. Although difficult to identify, genetic anomalies that predispose to particular reactions to anticancer therapies and physiological changes associated with complications can be used as biomarkers (Table 2).

### Nausea and vomiting

The most common symptoms following anti-cancer therapy are nausea and vomiting, which have been found to correlate with an increase in the hormone arginine vasopressin [193, 194]. Vasopressin induces vomiting by activation of chemoreceptor trigger zones in the area postrema of the brain, which induces afferent pathway activation of the medullary vomiting center [193]. No other markers predictive for vomiting or nausea are known.

### Mucositis

There is a need for readily accessible mucositis biomarkers. Current methods for symptom determination are invasive and harmful to patients undergoing concurrent anticancer treatments. Several diagnostic biomarkers for determining mucositis in chemotherapy patients have been generated including ^13^CO_2_, Lactulose/Mannitol, and citrulline; the latter has been found to be an accurate indicator of mucositis in HSCT patients [195, 196]. These tests assess components of mucositis-activating mechanisms that encourage tissue turnover in damaged gastrointestinal tracts. In chemotherapy patients, mucositis usually occurs in conjunction with diarrhea, and can be indicated by increases in the enzyme MMP-3, by changes in bacterial flora, such as increases in Bacteroides spp., and by inflammation in the gut mediated by an increase in fecal calprotectin [197]. A further complication that may occur during GI turnover is malabsorption; however, there are no specific biomarkers to indicate malabsorption in cancer patients. Stool weight can be used to indicate lack of absorption [198].

### Radiotherapy-related complications

Parotid gland [(18)F] fluorodeoxyglucose-labeled positron emission tomography-computed tomography (FDG-PET-CT) uptake has been found to be a possible biomarker for post-radiotherapy xerostomia [199]. In cervical cancer patients, radiotherapy is commonly performed in the pelvis, often resulting in mucosal dysfunction and numerous acute intestinal symptoms. Decreased levels of osteopontin (OPN) [200], a cytokine regulating mucosal protective functions through transduction pathways, and Transthyretin (TTR) [200], a transporter of thyroxine through the blood/brain barrier, have been associated with an increased chance of intestinal complications.

## FUTURE DIRECTIONS

Cancer patients suffer from cancer-induced and therapy- induced nutritional deficiencies (mainly protein and caloric). In patients with advanced cancers frequently, PCM advances to a serious and challenging problem, which is often not solved by increasing nutrient intake because it involves loss of lean body mass induced by metabolic alterations caused by the presence of the tumor. In patients with nutritional deficits at the time of diagnosis, treatments will likely aggravate side effects leading to suspension or decrease in dosage of therapy. To practice personalized cancer care, the clinic needs to monitor nutritional status of cancer patients right from diagnosis and throughout the treatment period. Currently, nutritional interventions are shown to have some beneficial effects on quality of life but there is little effect on mortality [201] and this minimal effect is likely caused by lack of early nutritional interventions. Research leading to more complete understanding of the relationship between complications of cancer treatment and progression toward malnutrition and acute nutritional deficits will allow for efficient measures to prevent the transition to cancer cachexia.

Nutritional supplementation offered in conjunction with patient selection at early stage of anti-cancer therapies can help patients withstand the toxic effects of therapy [202]. At onset and throughout the course of illness, patients should receive ongoing nutritional counseling. For selected patients, personalized dietary counseling with their family is important, and has been shown to improve the patient's nutritional status, decrease morbidity, and improve quality of life. Clinicians and healthcare providers should be equipped to educate the patient and family members on potential effects of a prescribed treatment regimen on nutrient intake, and should make dietary recommendations. Follow-up visits should include assessment of symptom control, weight, appetite, and function as part of continued medical surveillance and monitoring of health status. This level of care requires an interdisciplinary approach involving physicians, nurses, nutritionists, dietitians, and psychologists.

Research aimed at preventing and treating malnutrition must consider the multifactorial nature of the condition, beginning with establishment of a uniform definition of cancer-related malnourishment that should be followed as an eligibility criterion. In conjunction with nutritional therapy, a focus should be placed on management of symptoms resulting from toxicities associated with therapy. If therapy- associated nutritional complications get worsened prospects of postponing treatment until patient gets sufficient nourishment should be considered. Targeting specific symptoms with preventative measures, appropriate nutritional support, and drug administration will be beneficial to patient recovery. Considering the important role that changes in taste and smell exert on food intake and appetite, and in health and health-related quality of life of cancer patients, an objective assessment of qualitative taste and smell changes and adequate interventions should be applied. As symptoms of taste and smell disorders vary widely from patient to patient, specific interventions that combine pharmacologic and non-pharmacologic treatments into daily routines should be employed and customized for the individual patient.

Future directions in the field of cancer cachexia may also come from aggressive research efforts by scientists towards discovery and validation of “biomarkers” for early detection and prevention of cancer-induced weight loss. Research is also needed in the area of cancer-associated anorexia; reduced food intake is common among cancer patients, and effective therapies to improve appetite and daily caloric intake are lacking. Results from the study of candidate biomarkers that may predict possible physiological complications following anti-cancer therapy promise to facilitate effective management of treatment and to prevent progression toward cachexia.

## SUPPLEMENTARY TABLE


