# Supplementary material for: Protein calorie malnutrition, nutritional intervention and personalized cancer care
Source: Oncotarget. 2017 Feb 4;8(14):24009–30. doi: 10.18632/oncotarget.15103 (PMC5410360; doi:10.18632/oncotarget.15103)
Supplement: Supplementary file 1 [file oncotarget-08-24009-s001.docx]

**Supplementary Table 1**: **List of commonly prescribed drugs for the cancer- and chemotherapy-related symptoms.**

| **Symptom** | **Drug name (Generic)** | **Therapeutic Class** | **Pharmacological Target** | **Route**  **of administration** | **Other disease conditions or symptoms treated** | **Published clinical**  **trial citation** |
| --- | --- | --- | --- | --- | --- | --- |
| Nausea and Vomiting | Metoclopramide | Anti-emetic | Dopamine receptors antagonist | Oral  Injection | Loss of appetite  Dyspepsia | Wilson 2002 [[1](#_ENREF_1)] |
|  | Haloperidol | Anti-psychotic | Dopamine D2 receptor antagonist | Oral  Injection | Schizophrenia  Severe anxiety | Hardy 2010 [[2](#_ENREF_2)] |
|  | Cyclizine | Anti-cholinergic  Anti-histamine | Histamine H1 receptor antagonist | Oral  Intramuscular  Injection | Dizziness  Motion sickness | Johns 2006 [[3](#_ENREF_3)] |
|  | Domperidone | Anti-emetic | Dopamine receptors antagonist | Oral | Dyspepsia  Heartburn | Esseboom 1995 [[4](#_ENREF_4)] |
|  | Ondansetron | Anti-emetic  Anti-psychotic  Anti-anxiety | selective Serotonin 5-HT_3_ receptor antagonist | Oral  Intramuscular, Intravenous Injection | Postoperative nausea and vomiting | Polati 1997 [[5](#_ENREF_5)] |
|  | Scopolamine | Anti-muscarinic  Anti-cholinergic | Muscarinic acetylcholine receptors antagonist | Intramuscular, Intravenous,  Subcutaneous, Transdermal Injection | Irritable bowel syndrome  Urinary incontinence  Motion sickness | LeGrand 2010 [[6](#_ENREF_6)] |
|  | Prochlorperazine | Anti-psychotic | Dopamine receptor antagonist | Oral  Rectal  Intramuscular, Intravenous Injection | Schizophrenia  Vertigo | Hesketh 1997 [[7](#_ENREF_7)] |
|  | Chlorpromazine | Anti-psychotic | Dopamine receptors antagonist  Histamine H1 receptor antagonist | Oral  Intramuscular, Intravenous Injection | Intractable hiccups  Schizophrenia | Stuart-Harris 1983 [[8](#_ENREF_8)] |
|  | Methotrimeprazine | Anti-psychotic | Dopamine receptors antagonist | Oral | Schizophrenia  Bipolar disorder | Eisenchlas 2005 [[9](#_ENREF_9)] |
| Diarrhea | Loperamide hydrochloride | [Anti-diarrheal](http://www.drugbank.ca/mesh/antidiarrheals) | Opioid mu-receptor agonist  Non-selective calcium channel blocker | Oral | Chronic diarrhea associated with Inflammatory bowel disease or Gastroenteritis | Cascinu 2000 [[10](#_ENREF_10)] |
|  | Cholestyramine | Bile acid sequestrant | Bile acid sequestrant | Oral | Bile acid Diarrhea  Hypercholesterolemia | Flieger 2007 [[11](#_ENREF_11)] |
|  | Diphenoxylate | Anti-diarrheal | Opioid mu-receptor agonist | Oral | Chronic diarrhea  Psoriasis | Lustman 1987 [[12](#_ENREF_12)] |
|  | Octreotide Acetate | Anti-diarrheal | Somatostatin receptor agonist | Intravenous, Intramuscular, Subcutaneous Injection | Chronic diarrhea  Acromegaly | Topkan 2006 [[13](#_ENREF_13)] |
| Anorexia | Megestrol acetate | Appetite- stimulant  Synthetic progestational hormone | Binds to the progesterone receptor and  Neuropeptide Y (NPY) release in hypothalamus | Oral | Weight loss in AIDS  Recurrent/ metastatic breast, endometrial, prostate cancers  Cachexia | Tomíska 2003 [[14](#_ENREF_14)] |
|  | **Medroxyprogesterone acetate** | Appetite- stimulant  Synthetic progestational hormone | Binds to the Progesterone receptor and  Neuropeptide Y (NPY) release in hypothalamus | Oral  intramuscular, Subcutaneous Injection | Secondary amenorrhea Abnormal uterine bleeding  Endometriosis pain | Simons 1998 [[15](#_ENREF_15)] |
|  | Nandrolone  decanoate | Anabolic steroid | Androgen receptor agonist | Intramuscular Injection | Breast carcinoma  Antithrombin-III deficiency  HIV infection | Darnton 1999 [[16](#_ENREF_16)] |
|  | Oxandrolone | Anabolic steroid | Androgen receptor agonist | Oral | Weight loss following extensive surgery | Lesser 2008 [[17](#_ENREF_17)] |
| Cachexia | Dexamethasone | Corticosteroid | Glucocorticoid receptor agonist | Oral  Intramuscular, Intravenous  Injection | Allergic disorders  Skin conditions  Psoriasis  Breathing disorders | Loprinzi 1999 [[18](#_ENREF_18)] |
|  | Prednisolone | Corticosteroid | Glucocorticoid receptor agonist | Oral | Rheumatoid arthritis  Ankylosing spondylitis  Systemic lupus erythematosus | Lundholm 1994 [[19](#_ENREF_19)] |
|  | Methylprednisolone | Corticosteroid | Glucocorticoid receptor agonist | Oral  Intramuscular, Intravenous  Injection | Ulcerative colitis  Allergic disorders  Endocrine disorders | Della-Cuna1989 [[20](#_ENREF_20)] |
| Xerostomia | Amifostine | Cytoprotective Agent | Free radicals scavenger | Intravenous | Neutropenia-related fever  Mucositis  Renal toxicity | Gu 2014[[21](#_ENREF_21)] |
|  | Pilocarpine | Cholinergic agent | Muscarinic receptors agonist | Oral | Sjögrens syndrome | Fuertes-Cabero 2004 [[22](#_ENREF_22)] |
|  | Cevimeline | Cholinergic agent | Muscarinic receptors agonist | Oral | Sjogren's Syndrome | Chambers 2007 [[23](#_ENREF_23)] |
| Mucositis | Palifermin | Recombinant human keratinocyte growth factor (KGF) | Binds to human keratinocyte growth factor (KGF) receptor | Intravenous Injection | Mouth sores  Ulcers | Spielberger 2004 [[24](#_ENREF_24)] |
| Taste changes | Bovine Lactoferrin | Anti-bacterial  Immuno-stimulant | Induction of apoptosis Stimulation of cytokines production and activity of Immune cells and NK cells | Oral | Stomach and Intestinal ulcers  Diarrhea  Hepatitis C | Clinical trial ID:  NCT01596634  NCT01941810 |

REFERENCES

1. Wilson J, Plourde JY, Marshall D, Yoshida S, Chow W, Harsanyi Z, Pearen S and Darke A. Long-term safety and clinical effectiveness of controlled-release metoclopramide in cancer-associated dyspepsia syndrome: a multicentre evaluation. Journal of palliative care. 2002; 18(2):84-91.

2. Hardy JR, O'Shea A, White C, Gilshenan K, Welch L and Douglas C. The efficacy of haloperidol in the management of nausea and vomiting in patients with cancer. Journal of pain and symptom management. 2010; 40(1):111-116.

3. Johns RA, Hanousek J and Montgomery JE. A comparison of cyclizine and granisetron alone and in combination for the prevention of postoperative nausea and vomiting. Anaesthesia. 2006; 61(11):1053-1057.

4. Esseboom EU, Rojer RA, Borm JJ and Statius van Eps LW. Prophylaxis of delayed nausea and vomiting after cancer chemotherapy. The Netherlands journal of medicine. 1995; 47(1):12-17.

5. Polati E, Verlato G, Finco G, Mosaner W, Grosso S, Gottin L, Pinaroli AM and Ischia S. Ondansetron versus metoclopramide in the treatment of postoperative nausea and vomiting. Anesthesia and analgesia. 1997; 85(2):395-399.

6. Hinds MG, Welsh JH, Brennand DM, Fisher J, Glennie MJ, Richards NG, Turner DL and Robinson JA. Synthesis, conformational properties, and antibody recognition of peptides containing beta-turn mimetics based on alpha-alkylproline derivatives. Journal of medicinal chemistry. 1991; 34(6):1777-1789.

7. Hesketh PJ, Gandara DR, Hesketh AM, Edelman M, Webber LM, McManus M and Hainsworth JD. Improved control of high-dose-cisplatin-induced acute emesis with the addition of prochlorperazine to granisetron/dexamethasone. The cancer journal from Scientific American. 1997; 3(3):180-183.

8. Stuart-Harris R, Buckman R, Starke I and Wiltshaw E. Chlorpromazine, placebo and droperidol in the treatment of nausea and vomiting associated with cisplatin therapy. Postgraduate medical journal. 1983; 59(694):500-503.

9. Eisenchlas JH, Garrigue N, Junin M and De Simone GG. Low-dose levomepromazine in refractory emesis in advanced cancer patients: an open-label study. Palliative medicine. 2005; 19(1):71-75.

10. Earl RD. Dental claims processing. Texas dental journal. 1976; 94(6):36-38.

11. Flieger D, Klassert C, Hainke S, Keller R, Kleinschmidt R and Fischbach W. Phase II clinical trial for prevention of delayed diarrhea with cholestyramine/levofloxacin in the second-line treatment with irinotecan biweekly in patients with metastatic colorectal carcinoma. Oncology. 2007; 72(1-2):10-16.

12. Lustman F, Walters EG, Shroff NE and Akbar FA. Diphenoxylate hydrochloride (Lomotil) in the treatment of acute diarrhoea. The British journal of clinical practice. 1987; 41(3):648-651.

13. Topkan E and Karaoglu A. Octreotide in the management of chemoradiotherapy-induced diarrhea refractory to loperamide in patients with rectal carcinoma. Oncology. 2006; 71(5-6):354-360.

14. Tomiska M, Tomiskova M, Salajka F, Adam Z and Vorlicek J. Palliative treatment of cancer anorexia with oral suspension of megestrol acetate. Neoplasma. 2003; 50(3):227-233.

15. Simons JP, Schols AM, Hoefnagels JM, Westerterp KR, ten Velde GP and Wouters EF. Effects of medroxyprogesterone acetate on food intake, body composition, and resting energy expenditure in patients with advanced, nonhormone-sensitive cancer: a randomized, placebo-controlled trial. Cancer. 1998; 82(3):553-560.

16. Darnton SJ, Zgainski B, Grenier I, Allister K, Hiller L, McManus KG and Steyn RS. The use of an anabolic steroid (nandrolone decanoate) to improve nutritional status after esophageal resection for carcinoma. Diseases of the esophagus : official journal of the International Society for Diseases of the Esophagus / ISDE. 1999; 12(4):283-288.

17. Lesser G CD, Ottery F, et al. . (2008). A phase III randomized study comparing the effects of oxandrolone (Ox) and megestrol acetate (Meg) on lean body mass (LBM), weight (wt) and quality of life (QOL) in patients with solid tumors and weight loss receiving chemotherapy. ASCO MeetingProc Am Soc Clin Onc.

18. Loprinzi CL, Kugler JW, Sloan JA, Mailliard JA, Krook JE, Wilwerding MB, Rowland KM, Jr., Camoriano JK, Novotny PJ and Christensen BJ. Randomized comparison of megestrol acetate versus dexamethasone versus fluoxymesterone for the treatment of cancer anorexia/cachexia. Journal of clinical oncology : official journal of the American Society of Clinical Oncology. 1999; 17(10):3299-3306.

19. Lundholm K, Gelin J, Hyltander A, Lonnroth C, Sandstrom R, Svaninger G, Korner U, Gulich M, Karrefors I, Norli B and et al. Anti-inflammatory treatment may prolong survival in undernourished patients with metastatic solid tumors. Cancer research. 1994; 54(21):5602-5606.

20. Della Cuna GR, Pellegrini A and Piazzi M. Effect of methylprednisolone sodium succinate on quality of life in preterminal cancer patients: a placebo-controlled, multicenter study. The Methylprednisolone Preterminal Cancer Study Group. European journal of cancer & clinical oncology. 1989; 25(12):1817-1821.

21. Gu J, Zhu S, Li X, Wu H, Li Y and Hua F. Effect of amifostine in head and neck cancer patients treated with radiotherapy: a systematic review and meta-analysis based on randomized controlled trials. PloS one. 2014; 9(5):e95968.

22. Fuertes Cabero S, Setoain Perego X, Rovirosa Casino A, Mateos Fernandez JJ, Fuster Pelfort D, Ferre Jorge J, Navalpotro Yague V, Paredes Barranco P, Ortin Perez J, Muxi Pradas A and Pons Pons F. [Usefulness of pilocarpine in the prevention of xerostomia in patients with head and neck cancer treated with radiotherapy. Assessment with gammagraphy and salivary flow]. Revista espanola de medicina nuclear. 2004; 23(4):259-266.

23. Chambers MS, Posner M, Jones CU, Biel MA, Hodge KM, Vitti R, Armstrong I, Yen C and Weber RS. Cevimeline for the treatment of postirradiation xerostomia in patients with head and neck cancer. International journal of radiation oncology, biology, physics. 2007; 68(4):1102-1109.

24. Spielberger R, Stiff P, Bensinger W, Gentile T, Weisdorf D, Kewalramani T, Shea T, Yanovich S, Hansen K, Noga S, McCarty J, LeMaistre CF, Sung EC, Blazar BR, Elhardt D, Chen MG, et al. Palifermin for oral mucositis after intensive therapy for hematologic cancers. The New England journal of medicine. 2004; 351(25):2590-2598.
